# A Locally Aggressive Ameloblastic Fibro-Odontoma: A Case Report and Literature Review

**DOI:** 10.7759/cureus.20366

**Published:** 2021-12-12

**Authors:** Nivin Omar, Asad Ullah, Intisar Ghleilib, Nikhil Patel, Rafik A Abdelsayed

**Affiliations:** 1 Pathology, Medical College of Georgia, Augusta University, Augusta, USA; 2 Pathology and Laboratory Medicine, Medical College of Georgia, Augusta University, Augusta, USA; 3 Oral Pathology, Dental College of Georgia, Augusta, USA

**Keywords:** posterior maxilla, benign tumors, mixed odontogenic tumor, ameloblastic fibroma, ameloblastic fibro-odontoma

## Abstract

Ameloblastic fibro-odontoma (AFO) is a relatively rare, benign noninvasive mixed odontogenic neoplasm derived from epithelial and ectomesenchymal elements of the dental tissues. It usually presents with a mean age of 11.5 years and in the posterior segment of the mandible. It is extremely rare in the posterior maxilla. Although the latest WHO edition classified AFO as developing odontoma, here we present a locally aggressive AFO in a 21-year-old male involving the posterior maxilla and sinus with bone destruction. The patient presents with a two-year history of slowly progressive left facial swelling with malodorous drainage. The CT scan revealed a 5.5 x 4.3 cm well-circumscribed expansile mass with mixed attenuation and peripheral calcification occupying the left maxilla and sinus with bone destruction of the hard palate and orbital rim. According to the literature, most of the AFO cases were treated adequately through a conservative approach with just enucleation or surgical curettage. To our knowledge, our case is the first case treated aggressively with left maxillectomy, palatectomy, and reconstruction surgery because of its radiologic findings, which suggested a locally invasive neoplasm. Histologically, the specimen showed a mixture of proliferative epithelial, mesenchymal tissue elements, and variable amounts of mineralized deposits consisting of enamel matrix and dentinoid deposits, and the final diagnosis was AFO. In conclusion, we present a rare case of AFO with an unusual aggressive presentation, age group, and site involved. The radiographic, histopathologic features, and therapeutic approaches of this unusual locally aggressive tumor are presented with the review of relevant literature.

## Introduction

The abstract of this article was previously presented at the College of American Pathologist meeting, Chicago, September 25-28, 2021.

Ameloblastic fibro-odontoma (AFO) is a benign mixed odontogenic neoplasm that is to hamartoma [1]. In the latest World Health Organization (WHO) classification of head and neck tumors, AFO is classified as developing odontomas rather than a distinct tumor [2]. AFO represents about 2% of mixed odontogenic tumors and affects 3.4% of the population [3]. It is usually presented in people under 20 years of age with a mean of 11.5 years [4]. Clinically, this neoplasm presents as a painless slowly growing asymptomatic swelling and frequently associated with impacted tooth [4]. AFO most commonly affects the posterior mandible and is extremely rare in the maxilla [4]. It is noninvasive, but with rare recurrence according to most literature [4, 5]. Treatment is usually conservative with enucleation and curettage [6]. The purpose of this paper is to report an unusual case of a locally aggressive AFO affecting the left maxilla and sinus with a review of the relevant literature.

## Case presentation

A 21-year-old male with a two-year history of slowly progressive left maxillary and facial swelling presented to the emergency department for further evaluation. Associated symptoms included oral pain, facial pressure, and malodorous drainage. He had been treated with antibiotics and noticed the resolution of the drainage. Medical, social, and family histories were unremarkable, as were the results of a review of systems. There was no history of local trauma or infection.

The physical examination reveals a left facial swelling over the left maxillary sinus with intact dentition and mass effect on the left side of the palate. Also, there was a bulging of the hard and soft palate with extension into the vestibule.

**Figure 1:**
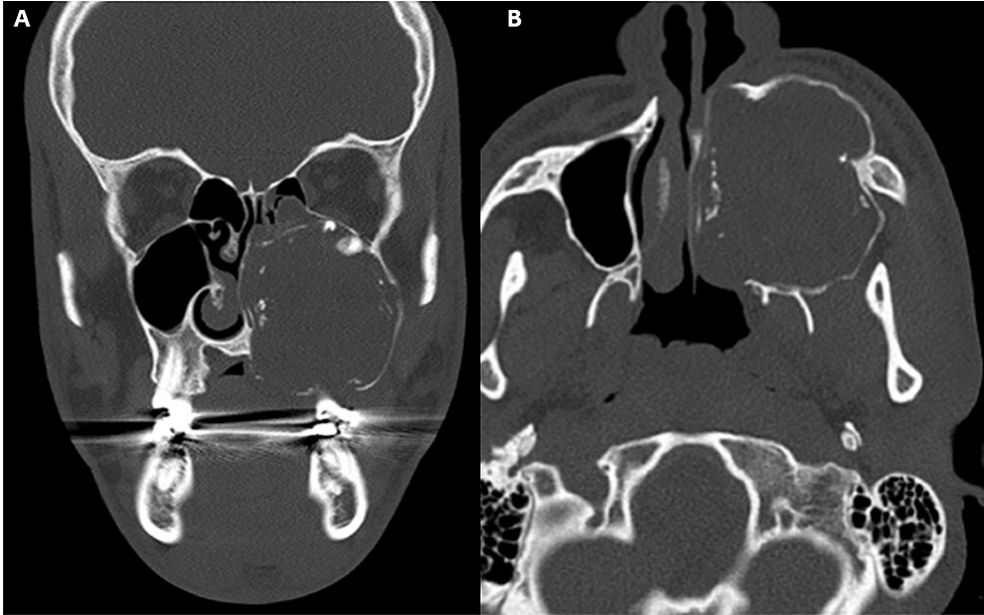
CT scan: Coronal (A) and axial (B) images showing expansile mixed lesion with predominant lucency with opaque foci and displaced maxillary molar tooth.

The CT scan revealed a 5.5 x 4.3 cm well-circumscribed expansile mass with mixed attenuation and peripheral calcification occupying the left side of maxilla and left sinus, with thinning and expansion of the bony walls, and destruction of the hard palate. The lesion extended into the left nasal cavity and abutted the left orbital floor. The tumor was associated with an unerupted tooth (Figures 1A, 1B).

Considering the clinical and radiographic examinations, a biopsy was indicated. Based on the small, submitted specimen, there were only mixed epithelial and ectomesenchymal elements without mineralized products. Consequently, a diagnosis of ameloblastic fibroma was made.

The case was discussed at the institution’s multidisciplinary head and neck tumor board and the treatment plan included maxillectomy, palatectomy with osteocutaneous free flap reconstruction under general anesthesia.

**Figure 2:**
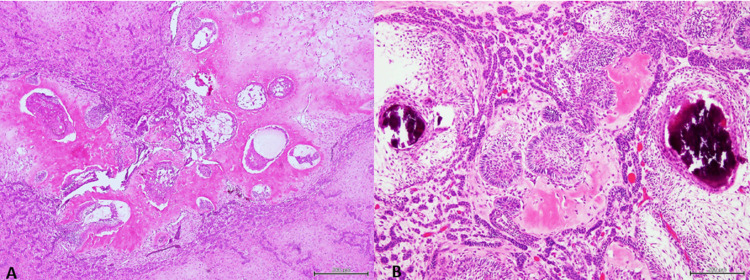
(A) H&E, 20x: Mixed proliferation of odontogenic epithelium and fibroblastic connective tissue interspersed by eosinophilic dentinoid matrix. (B) H&E, 20x: Ameloblastic follicles with cords of odontogenic epithelium and primitive fibroblastic tissue. Note basophilic enamel matrix juxtaposed to the ameloblastic epithelium.

Grossly, the lesion revealed a well-defined tan-white mass involving the posterior maxilla with impacted molar tooth and bone destruction. Microscopically, a well-circumscribed, non-encapsulated mixed odontogenic neoplasm consisted of epithelial and mesenchymal fibroblastic tissue elements. The neoplastic epithelial structures are present in the form of anastomosing cords and follicles with central polygonal and stellate-shaped cells surrounded by peripheral ameloblast-like columnar cells exhibiting nuclear palisading and reverse polarization. The epithelial cords and follicles are embedded in a moderately cellular fibroblastic tissue stroma with prominent ground substance imparting a myxoid appearance. No cellular atypia or mitosis was noted in neither the epithelial nor the fibroblastic tissues elements. Scattered foci of enamel matrix, characterized by its basophilic “fishscale” appearance were noted, some of which were juxtaposed to epithelial follicles surfaced by ameloblast-like cells. In other areas, hypocellular or acellular eosinophilic dentinoid matrix deposits were noted (Figures 2A, 2B). A final diagnosis of AFO was made. The patient underwent reconstructive surgery for his palatal defect, and currently being followed.

## Discussion

The new fourth edition of the WHO classification of head and neck tumors divides the odontogenic tumors into benign (epithelial, mesenchymal, and mixed) and malignant odontogenic tumors [2]. The etiology of these tumors is unknown, and most cases of benign tumors arise as de novo neoplasms [7]. Another change between the third and fourth editions is the classification of AFOs. In the third edition, AFOs were identified as a separate entity, classified as benign mixed odontogenic neoplasms. However, in the latest classification, they are considered a developing odontoma together with ameloblastic fibrodentinoma (AFD) [8]. Odontoma is a mixed epithelial and mesenchymal hamartomatous lesion with varying degrees of enamel and dentinoid matrix differentiation, but without cellular proliferation [3]. Odontomas are subdivided into compound and complex types [7]. Compound odontoma exhibits both morphologic differentiation into tooth-like structures and histologic differentiation into enamel and dentinoid deposits. On the other hand, complex odontoma exhibits only histologic differentiation into enamel and dentinoid deposits without morphologic differentiation into tooth-like structures, but rather shows these histologic elements intermixed in an amorphous mass. According to the literature, some authors support the idea that AFO is an immature complex odontoma and they are histologically indistinguishable [9,10]. This new classification may end the long debate mentioned in the literature about whether an AFO is its own unique entity or is a part of the histological changes in the development of complex odontoma [10,11]. Despite this new classification, some authors still argue that AFO and AFD are true neoplasms, and they do not support the idea of their maturation into odontoma [12].

To display the demographic presentation of AFO, a literature review of 108 AFO cases from 1967 to 2015 was used [13]. This review revealed the average age at presentation to be 6.3 years for males and 9.6 years for females. Only six of 108 cases presented after age 20. This makes our case rare since the patient in the current case presentation was 21 years old. Most of the reported case series showed no sex predilection [1]. However, in the aforementioned literature review of 108 cases, the male-to-female ratio was 1.62:1. Additionally, this review showed 89% of the cases were associated with an unerupted tooth, and the recurrence rate of lesions is 5.5%. The usual location for AFO is the posterior mandible, reported approximately in 60% of cases, followed by the rare involvement of the anterior maxilla [3]. In our case, the lesion was in the posterior maxilla involving the left maxillary sinus with an unerupted molar tooth and demonstrated excessive bone destruction. The lesion was average in size with 5.5 cm in the greatest dimension. The reported articles revealed a few cases of AFO that involved the maxilla [14,15]. According to most literature, AFO is a non-aggressive lesion [1,5]. Contrary to these findings, however, our case has the destruction of the left hard palate and orbital rim. Our literature review revealed one aggressive AFO case with cortical bone expansion [16] and another one with maxillary sinus bone destruction [17].

AFO is a benign, usually circumscribed noninvasive lesion [6]. Most of the reported cases were treated adequately through a conservative approach with just enucleation or surgical curettage accompanied by long-term follow-up [6]. Because it is a well-demarcated lesion, it can be easily separated from the surrounding bone [1]. Nevertheless, there is a controversy about either extracting the impacted tooth with conservative management or not [6]. Some authors support conservative management without removing the accompanying nonerupted teeth [5,18], while others favor the removal of the impacted molar tooth bud to prevent a recurrence. It is worth noting that, if there are histological changes in the recurrent lesion, like unorganized fibrous stroma and epithelial tissue loss, then a more aggressive approach with the removal of the impacted tooth is required to prevent recurrence [9,18] and malignant transformation [19]. Malignant transformation of AFO to odontogenic sarcoma is extremely rare [20]. To our knowledge, our case is the first case treated aggressively with left maxillectomy and reconstruction because of its radiologic findings, which suggested locally invasive neoplasm. There is one case in the literature that presented as a massive, 10 cm AFO with bone destruction that was treated with partial maxillectomy [17]. Our case provided a foundation to our knowledge that even if AFO is not massively large it can behave like a true neoplasm with bone destruction. A radical approach with reconstructive surgery is the preferred management for such locally aggressive odontogenic tumors because of the potential high recurrence rate and aggressive behavior [7].

## Conclusions

We report a rare case of a locally aggressive AFO, presented in the posterior maxilla and associated with an impacted molar tooth. The lesion is microscopically benign but demonstrated locally aggressive behavior and was treated with wide hemi-maxillectomy. Despite its benign nature, our case presented as an invasive neoplasm. Histopathological findings were consistent with AFOma without any malignant features. Clinicians should include AFO in the differential diagnosis when dealing with a mixed radiolucent-radiopaque mass in the maxilla associated with an unerupted tooth, even if presented as an invasive lesion in young people.
